# The study protocol for a quasi-experimental study on the effectiveness of a mobile health program in enhancing the physical and psychological capabilities of HIV voluntary counseling and testing among the deaf community

**DOI:** 10.12688/f1000research.161505.1

**Published:** 2025-02-26

**Authors:** Yafi Sabila Rosyad, Musheer Abdulwahid Aljaberi, Satheesh Babu Natarajan, Dely Maria

**Affiliations:** 1 Nursing, Lincoln University College, Petaling Jaya, Selangor, Malaysia; 2 STIKes Yogyakarta, Yogyakarta, Indonesia; 3 Applied Science, Lincoln University College, Petaling Jaya, Selangor, Malaysia; 4 Pharmacy, Lincoln University College, Petaling Jaya, Selangor, Malaysia; 5 Fakultas Vokasi, Universitas Kristen Indonesia, East Jakarta, Special Capital Region of Jakarta, Indonesia

**Keywords:** MHealth, HIV/AIDS, Deaf, VCT

## Abstract

**Background:**

Deaf person is risk population for health. During the covid-19 epidemic, deaf and hearing loss persons also suffer from psychological issues, post-traumatic stress disorder, and seropositive HIV.

**Objective:**

This study aims to examine the effectiveness of an mobile health educational program to increase mental health and HIV prevention among deaf community

**Methods:**

Quasi-experimental non-randomized controlled trial with single blinded participants, control standard therapy, assignment use parallel, purpose for health service research, study phase 2-3. pronounced to escalate the sample size to 40 deaf per group, which is 80 total participants.

**Results:**

The analysis of the data will be conducted utilizing the generalized estimation equation, with a confidence interval set at 95%. Significant differences, both between and within groups, will be identified at a threshold of P<.05. The findings of this study highlight the efficacy of a mobile educational program in enhancing mental health and preventing HIV within the deaf community. Furthermore, the outcomes of this research will augment existing knowledge regarding psychological distress, HIV prevention practices, and coping self-efficacy among individuals who are deaf.

**Conclusion:**

The intervention group is expected to demonstrate significantly lower scores in psychological distress during both the immediate evaluation and the assessment conducted three months post-intervention, compared to the wait-list group. Additionally, the intervention group is anticipated to exhibit enhanced levels of HIV prevention practices and coping self-efficacy, resulting in a greater degree of adjustment.

**Clinical trial:**

SLCTR/2024/039, 25 November 2024, https://slctr.lk/trials/slctr-2024-039

## Introduction

HIV prevention in the deaf-disabled population is one of the HIV-related health program’s concerns ( United Nations, 2016; World Health Organization (WHO), 2021b). Health initiatives relating to the promotion and prevention of sexual and reproductive health, particularly HIV illness, are often more accessible to those without disabilities. This is due to the fact that persons with impairments are seen as sexually inactive and thus get less attention from HIV initiatives ( Schenk et al., 2020). At the institutional level, the lack of knowledge and capacity of health workers on sexual and reproductive health issues, the negative attitude and lack of sensitivity of health workers, and the absence of privacy and accessible infrastructure for persons with disabilities are barriers that many people with disabilities encounter when attempting to access these services ( Schenk et al., 2020).

Persons with disabilities are 1.1 to 2.05 times more likely to engage in HIV-risk behaviors, such as substance misuse, alcoholism, sexual activity without the use of a condom, and partner switching. Awareness of HIV testing is also 1.1 times lower among people with disabilities compared to the general population ( Doyle et al., 2021). Interestingly, earlier research supporting the feasibility study indicate that only 28.9% of deaf individuals had undergone an HIV screening examination ( Olakunde & Pharr, 2020).

In addition to the absence of HIV-related information for the deaf, psychological issues are also a common obstacle. Hearing loss at any age is also associated with anxiety, low self-esteem and worth, cognitive decline, and diminished health-related quality of life as well as psychological distress ( Mehboob Khan et al., 2019). Adults and teenagers alike are at risk for very negative outcomes when they are experiencing psychological distress. The effect is a breakdown in social and psychological functioning ( Alika et al., 2016; Fergusson & Woodward, 2002).

Hearing loss was linked to distress in a major sample of persons under 70 years of age ( Bosdriesz et al., 2017; National Insitute on Aging (NIA), 2018). During the COVID-19 epidemic, deaf and hearing loss persons also suffer from psychological issues and post-traumatic stress disorder. The incidence of PTSD and depression among Hearing loss and hearing teens before to and during the COVID-19 epidemic in four Iranian cities (Borujerd, Malayer, Nahavand, ands Tuyskán). In our research, the prevalence of PTSD (46.43%) and depression (41.07%) among teenagers with hearing loss was much greater than predicted ( Ariapooran et al., 2021).

Their failure to establish good verbal communication may result in social rejection, a lack of education, and a poor work position, all of which have a significant negative influence on their self-esteem ( Gotowiec et al., 2022; Munoz-Baell & Ruiz, 2000; Strong & Shaver, 1991). The study of Jambor and Elliott (2005) on the self-esteem and coping methods of deaf students and deaf children indicated that deaf persons who identify with the deaf culture acquired higher self-esteem than those who identified with the hearing culture and involving physical appearance in hearing impaired (  Indiana et al., 2021; Jambor & Elliott, 2005; Theunissen et al., 2014).

According to WHO estimates, Over 5% of the world’s population, or 430 million individuals, have ‘disabling’ hearing loss and need rehabilitation (432 million adults and 34 million children). It is anticipated that by 2050, approximately 700 million individuals, or one in ten, would suffer from hearing impairment. Less than one percent of deaf, hard of hearing, and deaf and blind children in underdeveloped nations have access to school ( World Health Organization (WHO), 2021a). According to World Federation of the Deaf (WFD) data, 80% of deaf people are illiterate or poorly educated ( El-Soud & Hassan, 2009). Deaf individuals have difficulty understanding health recommendations ( Bahareh & Heidary, 2015). Due to their communication difficulties, limited understanding of deaf persons makes their health treatment more hard ( Harmer, 1999). According to research conducted by the England Mental Health Institute, there is a clear correlation between psychological diseases and hearing loss; the incidence of psychological issues among deaf children is almost double that of hearing children (40% against 25%). According to research conducted in several nations, psychiatric illnesses are manifestly more widespread among deaf individuals ( National Health Service, 2005). Even in the United States, less than 5% of deaf individuals get mental health treatment, and in the majority of impoverished nations, there is no mental health care for the deaf ( Joseph AM, 2009).

There are challenges for the deaf people to get health information ( Folkins et al., 2005). Deaf persons and their families need information and education to enhance general understanding of their condition. One of the educational components for deaf and hard of hearing individuals is the use of educational technology, such as computers and distant learning ( Kelly & McKenzie, 2018). Multimedia distant information and communication services may serve as the standard electronic platform for continuing deaf education ( Drigas et al., 2009).

Increasingly prevalent digital health technologies are employed for the prevention, diagnosis, and treatment of mental health issues. There is minimal research on mental health and HIV prevention in online initiatives for the deaf community. Engagement involves individual users’ ideas and emotions, level of activity, and opinions about technical features of the software, including characteristics of usability and attractiveness ( O’Brien & Toms, 2013). User engagement is also intimately tied to a program’s usability O’Brien & Toms (2013), which includes efficacy, efficiency, and user happiness ( The National Standards Authority of Ireland (NSAI), 2018).

Recent studies have begun to explore innovative approve to address these gaps. For instance, a 2023 study highlighted the effectiveness of digital health interventions in improving health literacy and self-efficacy among deaf individuals, demonstrating a positive impact on their overall well-being ( Arias López et al., 2023). The need cultural sensitive health communication strategies tailored to the deaf community to enhance engagement and understanding of health information in mental health ( Ulutorti, 2024).

This study aims to evaluate the effectiveness of mobile health educational program designed to enhance both mental health and HIV prevention among deaf individuals. By focusing on this underserved population, the research seeks to contribute valuable insights into the psychological distress, HIV prevention practice, and coping self-efficacy of deaf individuals, ultimately fostering a more inclusive approach to health care.

**Clinical trial: SLCTR/2024/039, 25 November 2024**, https://slctr.lk/trials/slctr-2024-039.

## Methods

The study used a quantitative method with a quasi-experimental non-randomized controlled trial approach. Data collection took three months from the first intervention given. The intervention group will be given education through the KaPi Program mobile health application using Indonesian sign language and the control group will be given education through e-books. To ensure that the intervention carried out is in accordance with the standards, the researcher using the Standard Protocol Items as a guide. The study will adhere to the Standard Protocol Items: Recommendations for Interventional Trials (SPIRIT) guidelines ( Chan et al., 2016), the Consolidated Standards of Reporting Trials (CONSORT) criteria ( Schulz et al., 2010), and the recommendations set forth by the Consolidated Standards of Reporting Trials of Electronic and Mobile Health Applications and Online Telehealth (CONSORT-EHEALTH) ( Eysenbach et al., 2011). Participation in the study was voluntary, and no financial compensation was offered. To ensure the accuracy and validity of this study, we will take strategic steps to minimize bias. The study will start with clear, testable objectives and hypotheses, and random sampling will be used to ensure representativeness. Data will be collected using valid, reliable instruments and standardized procedures. A blind or double-blind design will be implemented to reduce bias from both researchers and participants. Data analysis will follow appropriate statistical methods to avoid misinterpretation. The research process will be transparently reported, with methods and results available for replication. Peer review and potential replication by other researchers will further confirm the findings, ensuring the study produces valid, unbiased results.

### Study area

Yogyakarta district is a city in Indonesia that experiences a significant prevalence of HIV cases among the deaf population. The participants targeted for this study will be individuals associated with the Gerkatin NGO in Yogyakarta, Indonesia.

### Study design

**Figure 1.**
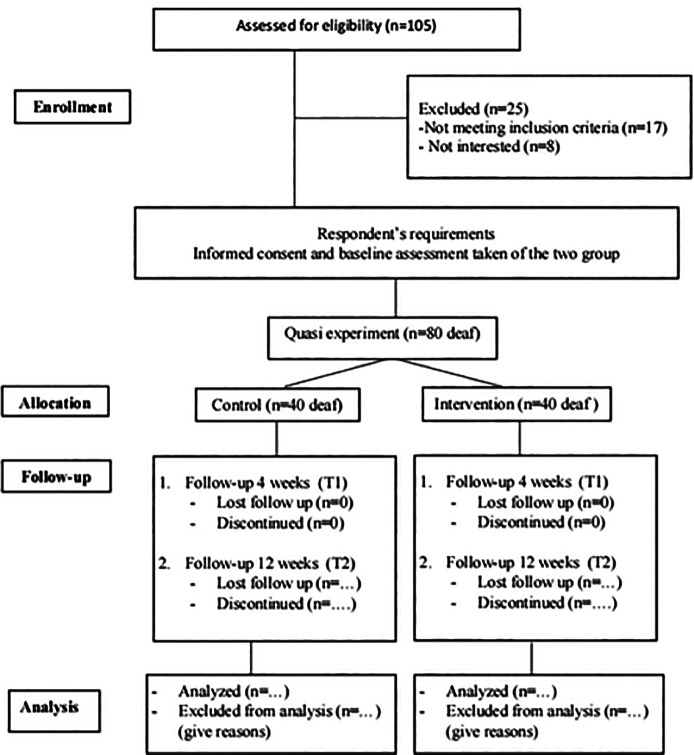
Summarized the study design.

Research design in this research will be use quasi-experimental non-randomized controlled trial with single blinded participants, control standard therapy, assignment use parallel, purpose for health service research, study phase 2-3. One or two (experimental group) receives the Mobile health KaPi Program intervention under test and the other (comparison group or control) receives the standard e book/leaflet. Then follow up on the two or more groups to see if there are any differences in the results. The results of the study and subsequent analysis are used to assess the effectiveness of the intervention mobile health application. Quasi-experimental are the most rigorous way to determine if there is a causal link between interventions and outcomes ( Polit and Beck, 2017).  Figure 1 provides an overview of the study design. The choice of this experimental design is grounded in its robustness and efficacy ( Creswell, 2016).

### Inclusion and exclusion criteria participants

Inclusion and exclusion criteria are clearly defined to ensure that the study population is representative of the target demographic. The study consisted of deaf Indonesian nationals 1. Age 18 to 65 years. 2. All gender (Male,Female, and other) 3. Sexually active 4. Has access to a smart phone. This study will exclude those who are deaf and 1. pregnant, 2. already diagnosed with HIV/AIDS, 3. illiterate 4. can’t speak Indonesian sign language. This careful selection process helps to control for confounding variables that could affect the outcomes, such as pre-existing health conditions or communication barriers ( Creswell, 2016; Hart et al., 2023). The significance of well-defined inclusion criteria in enhancing the internal validity of health research( Bodicoat et al., 2021; Patino & Ferreira, 2018).

### Recruitment

To assess eligibility, the researcher will initiate contact with all regional leaders of Gerkatin NGOs within the district, facilitated through the head of Gerkatin NGOs in the Yogyakarta area. A data collection permit letter, issued by the researcher’s affiliated university, will accompany this communication. This letter will be directed to the regional head of Gerkatin NGOs, who will subsequently disseminate it to all regional leaders. The recruitment strategy will incorporate various approaches known to enhance research participation, such as notifying the Gerkatin head about the study in advance, offering opportunities for involvement, visiting the Gerkatin representative, reaching out to potential participants via telephone and other communication methods, and allowing for inquiries to research staff regarding participation. Dedicated research personnel will oversee the recruitment process.

Following the dispatch of the letter and the acquisition of approval for data collection, the Gerkatin head or designated representative will reach out via telephone to verify eligibility according to the inclusion criteria and gauge interest in participating in the study. Information and consent forms will be provided to selected respondents to secure their agreement to participate. Should any respondents choose to withdraw during the research process, this will be permitted. All aspects related to the research process and the study’s duration will be thoroughly detailed in the Respondent Information and Consent Forms.

### Sample size

Sample size was calculated using the software Based on an a priori power analysis (G*Power) ( Faul et al., 2007). The sample size will be an *F*-test, an a priori type of power analysis ( Chow et al., 2002), with power (1- β) of 0.95, α level of 0.05, and an effect size at 0.20, for two groups. This formula provided a sample size of 66, which each group was 33 participants. Finally, the present study calculated a 20% ( In et al., 2020; Suresh & Chandrashekara, 2012) dropout rate (33 × 20% = 6.6, rounded up to 7) and pronounced to escalate the sample size to 40 deaf per group, which is 80 total participants.

### Outcome MHealth KaPi Program

Increase in physical and psychological capability of HIV voluntary counseling and testing

### Tools for collecting information in research

**Ebook Mental Health and HIV/AIDS**

The mental health and HIV/AIDS ebook contains general material related to mental health, psychological disorders, coping efficacy, and HIV/AIDS. The material in pdf can be accessed at https://doi.org/10.5281/zenodo.14784036 ( Rosyad, 2025a) *, * Data are available under the terms of the Creative Commons Zero “No rights reserved” data waiver (CC0 1.0 Public domain dedication).

**Mobile Health KaPi Program**

Mobile health KaPi program is education related about HIV/AIDS and mental. The proposed program will cover a period of 11 sessions and will be conducted over a period of 12.89 minute in each respondent of the intervention group. The program application can download at playstore with link https://play.google.com/store/apps/details?id=com.project.kapi. for the table this program can acces at https://doi.org/10.5281/zenodo.14784226 ( Rosyad, 2025b), Data are available under the terms of the Creative Commons Zero “No rights reserved” data waiver (CC0 1.0 Public domain dedication).

### Questionnaire to be utilized in this study

Researchers use Kessler Psychological Distress Scale (K10) from Kessler et al., (2002),is a 10-item questionnaire assessing anxiety and depressive symptoms over the past four weeks, with scores ranging from 10 to 50. A score under 20 suggests good mental health, while scores from 20 to 50 indicate varying levels of mental disorder severity ( Andrew & Slade, 2001; Kessler et al., 2002). Coping self-efficacy questionnaire will be adapting from Chesney et al. (2006), measuring confidence in coping behaviors, such as problem-focused coping and managing emotions. Respondents rate their confidence on an 11-point scale, and higher scores indicate greater coping self-efficacy, with good reliability and predictive validity for decreased psychological distress and increased well-being. and Knowledge, attitude, and practice HIV voluntary and counselling testing (K-A-P VCT) from Addis et al., (2013) is consists of 15 questions assessing participants’ knowledge, attitude, and practice regarding VCT services. Knowledge is measured through correct answers, attitudes are evaluated using a 5-item scale, and practice is determined by a single question about previous use of VCT services.

questionnaire will be adapting from Addis et al. (2013).

### Statistical analysis

Allocations of demographic information and predictors variables between groups were analyzed as a proportion (%) and case (n), respectively. Descriptive results like percentage and frequency distribution of all variables were presented using tables and charts. The c ^2^ and ANOVA analyzes were used to compare socioeconomic and demographic as well as baseline findings among the four groups, respectively. For the inferential statistic will be using the first, the association between each independent variable with the outcome variable was determined using binary logistic regression. The odds ratio, accompanied by the 95% confidence interval, was employed to evaluate the association. Additionally, multiple logistic regression analyses were conducted to account for the effects of confounding variables, utilizing a 95% confidence interval, with adjusted odds ratios serving as indicators of the strength of the association, where P<0.05 denotes statistical significance. Furthermore, generalized estimating equation (GEE) models, adhering to appropriate link function and distribution assumptions, were utilized to assess variations in findings over time and across four distinct groups. In summary, the models were calibrated to account for potential confounding factors.

## Results

The trial protocol of this study was approved by Health Research Ethics Committee Stikes Bethesda Yakkum, Indonesia have granted ethical approval No.036/KEPK.02.01/V/2023 and Trial registration: Sri Lanka Clinical Trials Registry (SLCTR) with number SLCTR/2024/039. Approval for participation in the study was secured from the governing bodies of the selected NGOs, Gerkatin. The findings of the study will be disseminated at both the cluster and individual levels, encompassing data on Psychological Distress, Coping Self-Efficacy, Knowledge, Attitudes, and Practices regarding HIV voluntary counseling and testing, intentions to withdraw from the study, the effectiveness of the intervention, estimated effect sizes along with their precision, and the primary outcomes. Preliminary results are anticipated to be submitted for publication by the conclusion of the 2024/2025 academic semester, and the research will be presented at both national and international conferences or published in a Scopus-indexed journal.

## Conclusion

This trial aims to offer significant insights into the implementation and effectiveness of the Mobile health KaPi program, an Android mobile application designed to enhance the physical and psychological capabilities of HIV voluntary counseling and testing within the deaf community, in comparison to the standard e-book. The outcomes of the process evaluation will deliver contextual information regarding the implementation decisions made for deaf individuals, the foundations of the deaf community, health workers, and health services.

### Ethics and consent

The trial protocol of this study was approved by head of ethics review committe Dwi Nugroho Heri Saputro, S.Kep., Ns., M.Kep., Sp.Kep.MB., PhD.NS on 05 November 2023, by Health Research Ethics Committee STIKES Bethesda Yakkum, Indonesia have granted ethical approval No.036/KEPK.02.01/V/2023 and Trial registration: Sri Lanka Clinical Trials Registry (SLCTR) with number SLCTR/2024/039 on 25 November 2024, https://slctr.lk/trials/slctr-2024-039.

Information and consent forms will be provided to selected respondents to secure their agreement to participate. There is no coercion to participate in this study, if the respondent agrees then the respondent will sign a written consent form to participate in the study. Should any respondents choose to withdraw during the research process, this will be permitted. All aspects related to the research process and the study’s duration will be thoroughly detailed in the Respondent Information and Consent Forms.

## Data Availability

No data associated with this article. Articles that report protocols for clinical trials adhere to the SPIRIT reporting guidelines https://doi.org/10.5281/zenodo.14762634 ( Rosyad et al., 2025), Data are available under the terms of the Creative Commons Zero “No rights reserved” data waiver (CC0 1.0 Public domain dedication).
