# A Quasi-Experimental Study Protocol on the Impact of a Mobile Health Program for HIV Counseling and Testing in the Deaf Community

**DOI:** 10.12688/f1000research.161505.3

**Published:** 2025-06-04

**Authors:** Yafi Sabila Rosyad, Musheer Abdulwahid Aljaberi, Satheesh Babu Natarajan, Dely Maria

**Affiliations:** 1 Department of Nursing, Faculty of Health Science, Universitas Bhakti Husada Indonesia, Kadugede Kuningan, West Java, Indonesia; 2 Nursing, Lincoln University College, Petaling Jaya, Selangor, Malaysia; 3 Applied Science, Lincoln University College, Petaling Jaya, Selangor, Malaysia; 4 Pharmacy, Lincoln University College, Petaling Jaya, Selangor, Malaysia; 5 Fakultas Vokasi, Universitas Kristen Indonesia, East Jakarta, Special Capital Region of Jakarta, Indonesia

**Keywords:** MHealth, HIV/AIDS, Deaf, VCT

## Abstract

**Background:**

Deaf persons are considered a high-risk population for health disparities. During the covid-19 epidemic, deaf persons also suffer from psychological issues, post-traumatic stress disorder, and seropositive HIV.

**Objective:**

This study aims to examine the effectiveness of an mobile health educational program to increase mental health and HIV prevention among deaf community

**Methods:**

This study employs a quasi-experimental design with a non-randomized controlled trial, involving single-blinded participants and a parallel group assignment, purpose for health service research, study phase 2-3. pronounced to escalate the sample size to 40 deaf per group, which is 80 total participants.

**Results:**

The analysis of the data will be conducted utilizing the generalized estimation equation, with a confidence interval set at 95%. Significant differences, both between and within groups, will be identified at a threshold of P<.05. The findings of this study highlight the efficacy of a mobile educational program in enhancing mental health and preventing HIV within the deaf community. Furthermore, the outcomes of this research will augment existing knowledge regarding psychological distress, HIV prevention practices, and coping self-efficacy among individuals who are deaf.

**Conclusion:**

The intervention group is expected to demonstrate significantly lower scores in psychological distress during both the immediate evaluation and the assessment conducted three months post-intervention, compared to the wait-list group. Additionally, the intervention group is anticipated to exhibit enhanced levels of HIV prevention practices and coping self-efficacy, resulting in a greater degree of adjustment.

**Clinical trial:**

SLCTR/2024/039, 25 November 2024, https://slctr.lk/trials/slctr-2024-039

## Introduction

HIV prevention in the deaf-disabled population is one of the HIV-related health program’s concerns ( United Nations, 2016; World Health Organization (WHO), 2021b). Health initiatives relating to the promotion and prevention of sexual and reproductive health, particularly HIV illness, are often more accessible to those without disabilities. This is due to the fact that persons with impairments are seen as sexually inactive and thus get less attention from HIV initiatives ( Schenk et al., 2020). At the institutional level, the lack of knowledge and capacity of health workers on sexual and reproductive health issues, the negative attitude and lack of sensitivity of health workers, and the absence of privacy and accessible infrastructure for persons with disabilities are barriers that many people with disabilities encounter when attempting to access these services ( Schenk et al., 2020).

Persons with disabilities are 1.1 to 2.05 times more likely to engage in HIV-risk behaviors, such as substance misuse, alcoholism, sexual activity without the use of a condom, and partner switching. Awareness of HIV testing is also 1.1 times lower among people with disabilities compared to the general population ( Doyle et al., 2021). Interestingly, earlier research supporting the feasibility study indicate that only 28.9% of deaf persons had undergone an HIV screening examination ( Olakunde & Pharr, 2020).

In addition to the absence of HIV-related information for the deaf, psychological issues are also a common obstacle. Hearing loss at any age is also associated with anxiety, low self-esteem and worth, cognitive decline, and diminished health-related quality of life as well as psychological distress ( Mehboob Khan et al., 2019). Adults and teenagers alike are at risk for very negative outcomes when they are experiencing psychological distress. The effect is a breakdown in social and psychological functioning ( Alika et al., 2016; Fergusson & Woodward, 2002).

Deaf persons was linked to distress in a major sample of persons under 70 years of age ( Bosdriesz et al., 2017; National Insitute on Aging (NIA), 2018). During the COVID-19 epidemic, deaf persons also suffer from psychological issues and post-traumatic stress disorder. The incidence of PTSD and depression among Hearing loss and hearing teens before to and during the COVID-19 epidemic in four Iranian cities (Borujerd, Malayer, Nahavand, ands Tuyskán). In our research, the prevalence of PTSD (46.43%) and depression (41.07%) among teenagers with hearing loss was much greater than predicted ( Ariapooran et al., 2021).

Their failure to establish good verbal communication may result in social rejection, a lack of education, and a poor work position, all of which have a significant negative influence on their self-esteem ( Gotowiec et al., 2022; Munoz-Baell & Ruiz, 2000; Strong & Shaver, 1991). The study of Jambor and Elliott (2005) on the self-esteem and coping methods of deaf students and deaf children indicated that deaf persons who identify with the deaf culture acquired higher self-esteem than those who identified with the hearing culture and involving physical appearance in hearing impaired (  Indiana et al., 2021; Jambor & Elliott, 2005; Theunissen et al., 2014).

According to WHO estimates, Over 5% of the world’s population, or 430 million individuals, have ‘disabling’ hearing loss and need rehabilitation (432 million adults and 34 million children). It is anticipated that by 2050, approximately 700 million individuals, or one in ten, would suffer from hearing impairment. Less than one percent of deaf, hard of hearing, and deaf and blind children in underdeveloped nations have access to school ( World Health Organization (WHO), 2021a). According to World Federation of the Deaf (WFD) data, 80% of deaf persons are illiterate or poorly educated ( El-Soud & Hassan, 2009). Deaf persons have difficulty understanding health recommendations ( Bahareh & Heidary, 2015). Due to their communication difficulties, limited understanding of deaf persons makes their health treatment more hard ( Harmer, 1999). According to research conducted by the England Mental Health Institute, there is a clear correlation between psychological diseases and hearing loss; the incidence of psychological issues among deaf children is almost double that of hearing children (40% against 25%). According to research conducted in several nations, psychiatric illnesses are manifestly more widespread among deaf persons ( National Health Service, 2005). Even in the United States, less than 5% of deaf individuals get mental health treatment, and in the majority of impoverished nations, there is no mental health care for the deaf ( Joseph AM, 2009).

There are challenges for the deaf person to get health information ( Folkins et al., 2005). Deaf persons and their families need information and education to enhance general understanding of their condition. One of the educational components for deaf and hard of hearing individuals is the use of educational technology, such as computers and distant learning ( Kelly & McKenzie, 2018). Multimedia distant information and communication services may serve as the standard electronic platform for continuing deaf education ( Drigas et al., 2009). The hearing health sector as a dynamic network shaped by innovation and regulation, ensuring quality and risk mitigation. Innovation included both technological and non-technological advancements benefiting consumers. Ethical alignment required consumer involvement in both processes to address stigma and reduce health disparities. ( Boisvert et al., 2024).

Increasingly prevalent digital health technologies are employed for the prevention, diagnosis, and treatment of mental health issues. There is minimal research on mental health and HIV prevention in online initiatives for the deaf community. Engagement involves individual users’ ideas and emotions, level of activity, and opinions about technical features of the software, including characteristics of usability and attractiveness ( O’Brien & Toms, 2013). User engagement is also intimately tied to a program’s usability O’Brien & Toms (2013), which includes efficacy, efficiency, and user happiness ( The National Standards Authority of Ireland (NSAI), 2018).

Recent studies have begun to explore innovative approve to address these gaps. For instance, a 2023 study highlighted the effectiveness of digital health interventions in improving health literacy and self-efficacy among deaf individuals, demonstrating a positive impact on their overall well-being ( Arias López et al., 2023). Educational interventions involving sign language interpretation and involvement of personnel involved in hearing loss resulted in significant increases in knowledge(Choi et al., 2023) The need cultural sensitive health communication strategies tailored to the deaf community to enhance engagement and understanding of health information in mental health ( Ulutorti, 2024).

Despite the proliferation of mobile health interventions in other populations, deaf persons remain underserved due to linguistic and cultural barriers. This study seeks to address that gap by evaluating a sign language-based mobile health program tailored to this community.

**Clinical trial: SLCTR/2024/039, 25 November 2024**, https://slctr.lk/trials/slctr-2024-039.

## Methods

This quasi-experimental study follows a non-randomized controlled trial design, in which participants are assigned to either the intervention group, receiving the mobile health KaPi program, and the control group, receiving standard educational materials in the form of e-books. To minimize potential bias, participants will be blinded to their group allocation; they will not be informed whether they are receiving the intervention or serving as part of the control group. Efforts will be made to ensure that the delivery of content appears comparable across groups to prevent participants from discerning their allocation. Data collection took three months from the first intervention given. To ensure that the intervention carried out is in accordance with the standards, the researcher using the Standard Protocol Items as a guide. The study will adhere to the Standard Protocol Items: Recommendations for Interventional Trials (SPIRIT) guidelines ( Chan et al., 2016), the Consolidated Standards of Reporting Trials (CONSORT) criteria ( Schulz et al., 2010), and the recommendations set forth by the Consolidated Standards of Reporting Trials of Electronic and Mobile Health Applications and Online Telehealth (CONSORT-EHEALTH) ( Eysenbach et al., 2011). Participation in the study was voluntary, and no financial compensation was offered. To ensure the accuracy and validity of this study, we will take strategic steps to minimize bias. The study will start with clear, testable objectives and hypotheses, and random sampling will be used to ensure representativeness. Data will be collected using valid, reliable instruments and standardized procedures. A blind or double-blind design will be implemented to reduce bias from both researchers and participants. Data analysis will follow appropriate statistical methods to avoid misinterpretation. The research process will be transparently reported, with methods and results available for replication. Peer review and potential replication by other researchers will further confirm the findings, ensuring the study produces valid, unbiased results.

### Study area

Yogyakarta district is a city in Indonesia that experiences a significant prevalence of HIV cases among the deaf population. The participants targeted for this study will be individuals associated with the Gerkatin NGO in Yogyakarta, Indonesia.

### Study design

**Figure 1.**
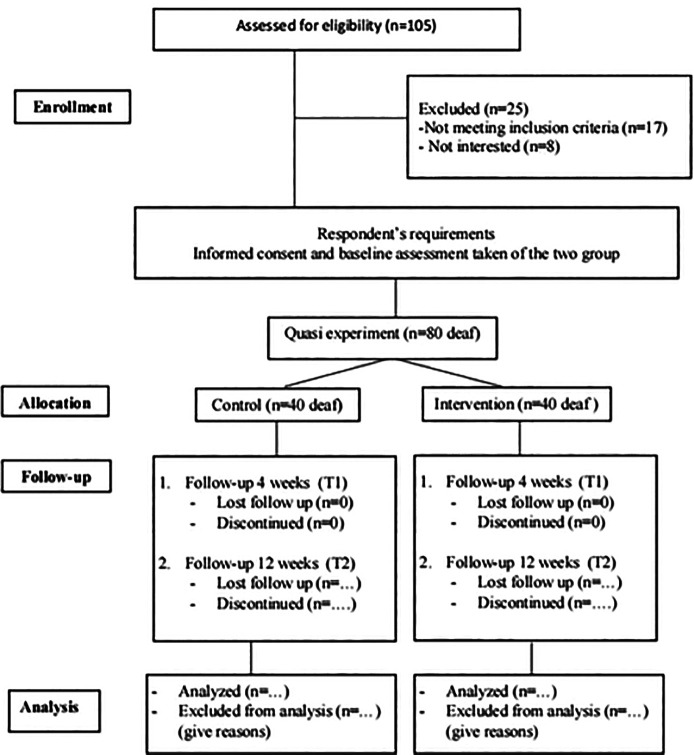
Summarized the study design.

This study will employ a quasi-experimental, non-randomized controlled trial design with single blinding applied to participants. The control group will receive standard therapy, while the intervention group will receive the mobile health program. A parallel assignment approach will be used, reflecting the structure of health services research. This study corresponds to a Phase 2–3 trial, aiming to evaluate both the feasibility and preliminary effectiveness of the intervention. One or two (experimental group) receives the Mobile health KaPi Program intervention under test and the other (comparison group or control) receives the standard e book/leaflet. Then follow up on the two or more groups to see if there are any differences in the results. The results of the study and subsequent analysis are used to assess the effectiveness of the intervention mobile health application. Quasi-experimental are the most rigorous way to determine if there is a causal link between interventions and outcomes ( Polit and Beck, 2017).  Figure 1 provides an overview of the study design. The choice of this experimental design is grounded in its robustness and efficacy ( Creswell, 2016).

### Inclusion and exclusion criteria participants

Inclusion and exclusion criteria are clearly defined to ensure that the study population is representative of the target demographic. The study consisted of deaf Indonesian nationals 1. Age 18 to 65 years. 2. All gender (Male, Female, and other) 3. Sexually active 4. Has access to a smart phone. This study will exclude those who are deaf and 1. pregnant, 2. already diagnosed with HIV/AIDS, 3. illiterate 4. can’t speak Indonesian sign language. This careful selection process helps to control for confounding variables that could affect the outcomes, such as pre-existing health conditions or communication barriers ( Creswell, 2016; Hart et al., 2023). The significance of well-defined inclusion criteria in enhancing the internal validity of health research ( Bodicoat et al., 2021; Patino & Ferreira, 2018).

### Recruitment

To assess eligibility, the researcher will initiate contact with all regional leaders of Gerkatin NGOs within the target district, facilitated through coordination with the head of Gerkatin in the Yogyakarta area. This process will be accompanied by a formal data collection permit issued by the researcher’s affiliated university. The letter will be forwarded to the regional Gerkatin head, who will then distribute it to local branch leaders to support coordinated recruitment.

The recruitment strategy will apply multiple approaches commonly used to enhance participation in research, such as early notification to Gerkatin leadership, offering involvement opportunities, on-site visits to Gerkatin representatives, outreach via phone and digital communication, and allowing potential participants to consult with research staff regarding study participation. Dedicated research personnel will supervise and document the recruitment process.

Once permission is obtained, the Gerkatin head or a designated representative will contact eligible individuals via telephone to assess inclusion criteria and confirm their willingness to participate. Eligible participants will be provided with detailed information and consent forms, including explanations in Indonesian Sign Language, and asked to sign written consent forms.

To support participant retention throughout the three-month study period, a dedicated WhatsApp group (WAG) will be created for enrolled participants. This group will serve as a monitored communication platform for sending reminders, providing support, and addressing questions related to the intervention and follow-up activities. Periodic check-ins and app-based notifications will also be used to promote sustained engagement. Participants will be reminded of their right to withdraw from the study at any time without penalty. All study procedures and timelines will be clearly explained in the Respondent Information and Consent Forms.

To address potential technological barriers, the study will ensure that all participants have access to a smartphone compatible with the KaPi mobile application. For participants without a suitable device, the research team will collaborate with local Gerkatin branches to provide access to shared devices or temporary loaner phones during the study period. In addition, brief orientation sessions will be conducted in Indonesian Sign Language to improve digital literacy and familiarize users with app features. If participants experience limited or unstable internet connectivity, the mobile app is designed to support offline functionality, allowing them to access core content without continuous internet access. These measures aim to minimize dropout due to technical challenges and ensure inclusive participation.

### Sample size

The sample size was determined using the software Based on an a priori power analysis (G*Power 3.1) ( Faul et al., 2007). An *F*-test (ANOVA: repeated measures, within-between interaction) was selected as the statistical test, assuming two groups (intervention and control). The analysis used a conventional alpha level (α) of 0.05, a statistical power (1 - β) of 0.95, and a small to medium effect size (f = 0.20), in line with guideline from ( Chow et al., 2002). Based on the parameters, the minimum required sample size was calculated to be 66 participants (33 per group).

 To account for potential attrition, a 20% dropout rate was anticipated, as supported by previous studies ( In et al., 2020; Suresh & Chandrashekara, 2012). The adjustment led to an additional 7 participants per group, resulting in a final target sample size of 40 participants per group, or total of 80 deaf participants for this study. The final analysis will be conducted using Generalized Estimating Equations (GEE) to account for the correlated nature of repeated measures over time and provide robust estimates of intervention effects.

### Outcome MHealth KaPi Program

Increase in physical and psychological capability of HIV voluntary counseling and testing.

### Tools for collecting information in research

**Ebook Mental Health and HIV/AIDS**

The mental health and HIV/AIDS ebook contains general material related to mental health, psychological disorders, coping efficacy, and HIV/AIDS. The material in pdf can be accessed at https://doi.org/10.5281/zenodo.14784036 ( Rosyad, 2025a) *, * Data are available under the terms of the Creative Commons Zero “No rights reserved” data waiver (CC0 1.0 Public domain dedication).

**Mobile Health KaPi Program**

The KaPi mobile health program consists of 11 structured sessions, each lasting approximately 12 minutes. These sessions cover topics such as HIV prevention strategies, coping mechanisms for psychological distress, and self-efficacy improvement, all delivered through Indonesian sign language videos within the app. The program application can download at playstore with link https://play.google.com/store/apps/details?id=com.project.kapi. for the table this program can acces at https://doi.org/10.5281/zenodo.14784226 ( Rosyad, 2025b), Data are available under the terms of the Creative Commons Zero “No rights reserved” data waiver (CC0 1.0 Public domain dedication).

### Questionnaire to be utilized in this study

Researchers use Kessler Psychological Distress Scale (K10) from Kessler et al., (2002), is a 10-item questionnaire assessing anxiety and depressive symptoms over the past four weeks, with scores ranging from 10 to 50. A score under 20 suggests good mental health, while scores from 20 to 50 indicate varying levels of mental disorder severity ( Andrew & Slade, 2001; Kessler et al., 2002). Coping self-efficacy questionnaire will be adapting from Chesney et al. (2006), measuring confidence in coping behaviors, such as problem-focused coping and managing emotions. Respondents rate their confidence on an 11-point scale, and higher scores indicate greater coping self-efficacy, with good reliability and predictive validity for decreased psychological distress and increased well-being. and Knowledge, attitude, and practice HIV voluntary and counselling testing (K-A-P VCT) from Addis et al., (2013) is consists of 15 questions assessing participants’ knowledge, attitude, and practice regarding VCT services. Knowledge is measured through correct answers, attitudes are evaluated using a 5-item scale, and practice is determined by a single question about previous use of VCT services.

Questionnaire will be adapting from Addis et al. (2013).

### Statistical analysis

Demographic characteristics and predictor variables were summarized as frequencies (n) and percentages (%) for categorical data, and means with standard deviations for continuous data. Descriptive statistics, including percentage and frequency distributions, are presented using tables and charts. Chi-square tests and independent t-tests (or ANOVA where appropriate) were used to compare socioeconomic and baseline characteristics between the two study groups.

For inferential analysis, the association between each independent variable and the outcome variable was initially examined using binary logistic regression. The primary outcomes—psychological distress (K10), HIV prevention knowledge and practices (KAP VCT), and communication self-efficacy (CSE)—were analyzed using Generalized Estimating Equations (GEE) to account for repeated measures and within-subject correlations over time. The GEE model included main effects of group and time, as well as their interaction term (group × time), to evaluate differential changes between intervention and control groups across three measurement points (baseline, post-intervention, and 4-week follow-up). Adjusted odds ratios with 95% confidence intervals are reported.

Missing data will be handled under the missing at random (MAR) assumption inherent in GEE, and sensitivity analyses will be conducted to assess the robustness of findings.

## Results

The trial protocol of this study was approved by the Health Research Ethics Committee of STIKes Bethesda Yakkum, Indonesia, with ethical approval No. 036/KEPK.02.01/V/2023. Trial registration was obtained through the Sri Lanka Clinical Trials Registry (SLCTR) under number SLCTR/2024/039. Approval for participation in the study was also secured from the governing bodies of the selected NGOs, including Gerkatin.

The findings of the study will be disseminated at both the cluster and individual levels. These will include results related to Psychological Distress, Coping Self-Efficacy, Knowledge, Attitudes, and Practices regarding HIV Voluntary Counseling and Testing (VCT), participant retention, intervention effectiveness, estimated effect sizes and their confidence intervals, and all designated primary outcomes.

To ensure accessibility and community impact, results will be communicated to the deaf community through culturally appropriate channels, including the production of summary videos in Indonesian Sign Language. These videos will explain key findings in accessible formats and will be distributed via social media platforms, Gerkatin community groups, and through the KaPi mobile application. In addition, in-person or virtual community feedback sessions will be organized in collaboration with local Gerkatin chapters to promote dialogue, reflection, and community engagement around the findings.

Preliminary results are anticipated to be submitted for publication by the conclusion of the 2024/2025 academic semester, and the research will also be presented at both national and international conferences or published in a Scopus-indexed journal.

## Conclusion

This study aims to provide evidence on the feasibility and effectiveness of a mobile health intervention tailored for deaf individuals, potentially informing future public health interventions and digital health strategies.

### Deaf person is risk population for health ethics and consent

The trial protocol of this study was approved by head of ethics review committe Dwi Nugroho Heri Saputro, S.Kep., Ns., M.Kep., Sp.Kep.MB., PhD.NS on 05 November 2023, by Health Research Ethics Committee STIKES Bethesda Yakkum, Indonesia have granted ethical approval No.036/KEPK.02.01/V/2023 and Trial registration: Sri Lanka Clinical Trials Registry (SLCTR) with number SLCTR/2024/039 on 25 November 2024, https://slctr.lk/trials/slctr-2024-039.

All participants will be provided with detailed study information delivered in Indonesian Sign Language to ensure accessibility for deaf individuals. To confirm their understanding, comprehension questions and interactive elements will be used before obtaining informed consent. Informed consent will be obtained through a combination of video-recorded agreements using sign language and a signed written consent form as formal documentation.

Participation is completely voluntary with no coercion. Participants have the right to withdraw at any time without penalty, and this will be clearly communicated during the consent process.

Data collected via the mobile application will be securely stored on encrypted servers accessible only to authorized research staff. Participant anonymity will be maintained by using unique codes, and no identifiable information will be published. Procedures for protecting data privacy and for withdrawal—including deletion of data upon participant request—are clearly detailed in the Respondent Information and Consent Forms.

## Data Availability

No data associated with this article. Articles that report protocols for clinical trials adhere to the SPIRIT reporting guidelines https://doi.org/10.5281/zenodo.14762634 ( Rosyad et al., 2025), Data are available under the terms of the Creative Commons Zero “No rights reserved” data waiver (CC0 1.0 Public domain dedication).
