# Supplementary material for: A Quasi-Experimental Study Protocol on the Impact of a Mobile Health Program for HIV Counseling and Testing in the Deaf Community
Source: F1000Res. 2025 Jun 4;14:239. Originally published 2025 Feb 26. [Version 3] doi: 10.12688/f1000research.161505.3 (PMC12487785; doi:10.12688/f1000research.161505.3)
Supplement: Peer review history

# Reviewer report: Reviewer response for version 3

**DOI:** 10.5256/f1000research.183248.r417677

**Published:** 2025-09-27

**Authors:** Artur Acelino Francisco Luz Nunes Queiroz

**Affiliations:** 1 Florida State University, Tallahassee, Florida, USA

A Quasi-Experimental Study Protocol on the Impact of a Mobile Health Program for HIV Counseling and Testing in the Deaf Community

 Background

 The background section of the paper is robust and well supported by the network of literature that is relevant. However I believe it could benefit from a more streamlined and focus approach. The authors use many of the space talking about causes and consequences of hearing loss, and the implications of the COVID pandemic, without a clear connection to the actual intervention they are testing.

 Methods

 Some sections of the methods are written in future tense and others in past tense what makes it confusing to follow.

 Whatsapp group could cause a contamination effect?

 The outcome is unclear: Increase in physical and psychological capability of HIV voluntary counseling and testing.

 I believe that a theoretical and operational explanation of it would make the study clearer and stronger.

 The questionnaires described do not reflect the outcomes, specially the physical aspects.

 What is the relationship between mental health, physical capacity and HIV prevention?

Is the study design appropriate for the research question?

Partly

Is the rationale for, and objectives of, the study clearly described?

Partly

Are sufficient details of the methods provided to allow replication by others?

Partly

Are the datasets clearly presented in a useable and accessible format?

Not applicable

Reviewer Expertise:

HIV prevention and digital health.

I confirm that I have read this submission and believe that I have an appropriate level of expertise to confirm that it is of an acceptable scientific standard, however I have significant reservations, as outlined above.

# Reviewer report: Reviewer response for version 3

**DOI:** 10.5256/f1000research.183248.r389795

**Published:** 2025-06-16

**Authors:** Pakorn Akkakanjanasupar

**Affiliations:** 1 Chulalongkorn University, Bangkok, Thailand

**Assessment of the Revisions**

 It's clear the author has truly taken to the feedback and put significant effort into refining this manuscript.

 The proposed revisions effectively enhance both the clarity and scientific rigor of the protocol. From sharpening the title and abstract to providing crucial details in the methodology and ethical considerations, every change seems well-considered. Their commitment to a thorough language review is also a vital step that will ensure the document is polished and professional.

 Overall, these are excellent adjustments. They promise to transform this protocol into a much stronger and more impactful contribution to the field.

Is the study design appropriate for the research question?

Yes

Is the rationale for, and objectives of, the study clearly described?

Partly

Are sufficient details of the methods provided to allow replication by others?

Partly

Are the datasets clearly presented in a useable and accessible format?

Not applicable

Reviewer Expertise:

My review, based on my public health and digital health research background, focused on the manuscript's rationale, design, ethics (for marginalized groups), and intervention accessibility/cultural sensitivity. The authors' revisions have successfully improved the study's methodological clarity, cultural competence, and practical implementation for digital health equity.

I confirm that I have read this submission and believe that I have an appropriate level of expertise to confirm that it is of an acceptable scientific standard.

# Reviewer report: Reviewer response for version 2

**DOI:** 10.5256/f1000research.179725.r380081

**Published:** 2025-05-27

**Authors:** Pakorn Akkakanjanasupar

**Affiliations:** 1 Chulalongkorn University, Bangkok, Thailand

**Summary:**

 Thank you for the opportunity to review this important and much-needed study. The proposed research addresses a significant gap in health equity by focusing on the deaf community—an underserved population often excluded from conventional health interventions. The integration of a mobile health (mHealth) platform using sign language-based video education is both innovative and contextually appropriate. However, to strengthen the scientific rigor, clarity, and ethical robustness of this study protocol, several areas require refinement.

 The comments provided below are intended to support the authors in improving the transparency, replicability, and overall quality of the manuscript in line with academic standards and research ethics.

***General Assessment​​​​​​ & Suggestions***

*1. Rationale and Objectives (Partly)*

 The manuscript presents a well-intentioned public health intervention. The introduction offers useful background, including relevant epidemiological data and challenges faced by the deaf population. However, the rationale is not explicitly or succinctly stated. The research problem and the gap in existing literature, particularly regarding the limited evidence on mobile-based HIV prevention in the deaf community, needed clearer articulation.

 I recommend adding a concise statement in the introduction that directly links the identified public health challenge to the need for this specific study. A sentence such as, *“Despite the proliferation of mobile health interventions in other populations, deaf individuals remain underserved due to linguistic and cultural barriers. This study seeks to address that gap by evaluating a sign language-based mobile health program tailored to this community,”* would sharpen the focus and purpose of the research.

*2. Study Design and Intervention (Yes)*

 A quasi-experimental design is appropriate given the practical constraints in randomizing this specific population. The use of a single-blinded structure also aligns with the implementation model. However, the explanation of blinding, control conditions, and assignment procedures is underdeveloped.

 It would strengthen the protocol to include a detailed explanation of the blinding process. For example, if outcome assessors are blinded to group allocation, this should be explicitly stated, along with procedures to maintain that blinding. Additionally, since allocation is not randomized, the authors should explain how potential biases will be mitigated—for instance, by using matched groups or adjusting for baseline differences in the analysis.

*3. Methodology and Replicability (Partly)*

 The manuscript includes appropriate tools (K10, CSE, KAP VCT) and a valid analytical approach (GEE). However, the intervention description and sampling calculations are vague.

 I recommend that the authors include a session-by-session overview of the KaPi program, either in the main text or as an appendix. Each session’s objectives, thematic content, and format (e.g., video, quiz) should be described. Regarding sample size, while G*Power is correctly cited, the effect size and statistical assumptions used in the calculation (e.g., α = 0.05, power = 0.95) should be stated clearly. This transparency is essential for methodological integrity.

*4. Statistical Analysis (Partly)*

 The use of Generalized Estimation Equations (GEE) is methodologically sound for analyzing repeated measures. Nonetheless, the statistical section is overly verbose, with some inconsistencies (e.g., reference to “four groups” instead of two).

 It should specify the primary and secondary outcomes, the time points of measurement, and the interaction terms to be tested. Also, clarify how missing data will be handled and ensure that references to group structure are consistent with the described design. I encourage the authors to revise this section for clarity and conciseness.

*5. Ethical Considerations and Accessibility (Needs Strengthening)*

 The manuscript notes that consent will be obtained via sign language video agreements, which is commendable. However, further detail is needed on how informed consent will be verified and how data privacy is protected, especially given the use of a mobile application.

 It is better if the authors could describe how participant understanding of the study will be verified (e.g., through comprehension questions or interactive elements). It is also important to discuss how data from the mobile app will be stored and protected, particularly regarding participant anonymity and consent withdrawal. This section should also affirm that no coercion will be used and that participants may exit the study at any point without penalty.

*6. Language and Presentation (Requires Revision)*

 The manuscript is significantly hindered by grammatical errors, awkward syntax, and inconsistent terminology (e.g., “deaf person,” “hearing loss individuals”). This affects the clarity and professionalism of the protocol.

 A comprehensive language review is strongly advised. This should address syntax, grammar, and word choice. Terms referring to the study population should be standardized throughout the manuscript; for example, consistently using “deaf individuals” instead of alternating with “deaf persons” or “hearing loss individuals.” This will enhance the manuscript’s coherence and respect for community-preferred terminology.

*7. Additional Comments*

7.1 Theoretical Framework: Identify a theoretical model guiding the intervention (e.g., Social Cognitive Theory), to support the focus on behavior change and self-efficacy.

 I encourage the authors to specify a behavioral or educational theory (e.g., Health Belief Model, Social Cognitive Theory) that informs the intervention’s focus on self-efficacy and behavioral change. This theoretical anchoring would enhance the scientific robustness of the intervention design.

7.2 Recruitment and Retention: Expand on how participants will be retained over three months. Describe follow-up strategies or engagement features within the app.

 The authors should expand on how they will ensure sustained participation over the three-month study period. This might include periodic check-ins, app-based reminders, or participant incentives.

7.3Technological Barriers: Address potential challenges in device access, digital literacy, and internet connectivity. Propose solutions or alternatives.

 A brief discussion of how the study will address potential technological barriers—such as smartphone access, digital literacy, and internet connectivity—would strengthen the feasibility of the protocol.

7.4 Dissemination Plan: Indicate how results will be communicated to the deaf community (e.g., videos in sign language, community briefings).

 The manuscript should include a clear plan for sharing study findings with the deaf community, such as through sign language videos or community feedback sessions.

**Conclusion**

 This is a socially impactful and methodologically promising study addressing a population often excluded from digital health innovations. The intervention is both culturally sensitive and contextually relevant. However, to meet indexing standards, the manuscript requires revisions in methodological clarity, ethical detail, and language quality.

**Final Recommendation**: 

 With these improvements, the protocol will make a meaningful contribution to health equity, HIV prevention, and inclusive digital health research. I commend the authors for their commitment to addressing disparities in deaf health care and look forward to seeing this work developed further.

Is the study design appropriate for the research question?

Yes

Is the rationale for, and objectives of, the study clearly described?

Partly

Are sufficient details of the methods provided to allow replication by others?

Partly

Are the datasets clearly presented in a useable and accessible format?

Not applicable

Reviewer Expertise:

As a reviewer with a background in public health research, digital health intervention design, and applied research methodology, my evaluation of this manuscript focuses on several key domains: the clarity and relevance of the study rationale, the appropriateness and transparency of the study design, the ethical considerations in working with a marginalized population, and the accessibility and cultural sensitivity of the proposed mobile health intervention.In particular, I have assessed:The study’s rationale and objectives, with attention to the public health implications and population-specific context;The quasi-experimental design, including its alignment with the research aims and appropriateness for the study population;The description of the intervention and its accessibility through sign language and mobile platforms;The ethical framework, particularly related to informed consent procedures for deaf participants and data privacy concerns;The community engagement and dissemination plans, given the importance of participatory approaches in underserved populations.While I have reviewed the statistical methods and instruments used for data collection, I defer detailed evaluation of advanced statistical modeling and HIV-specific clinical outcomes to reviewers with specialized expertise in those areas. My feedback is intended to support the authors in enhancing the study’s methodological clarity, cultural competence, and practical implementation for digital health equity.

I confirm that I have read this submission and believe that I have an appropriate level of expertise to confirm that it is of an acceptable scientific standard, however I have significant reservations, as outlined above.

# Reviewer report: Reviewer response for version 1

**DOI:** 10.5256/f1000research.177544.r369983

**Published:** 2025-03-14

**Authors:** Isiko Isaac

**Affiliations:** 1 Department of Community Medicine, Axel Pries Institute of Public Health and Biomedical Sciences, NIMS University, Jaipur, Rajasthan, India; 2 School of Medicine, Makerere University, Kampala, Central Region, Uganda

**Dear Editor,**

 Thank you for the opportunity to review this manuscript on the effectiveness of a mobile health program for HIV counseling and testing among the deaf community. Your study addresses a crucial public health issue with significant potential impact.

 While the manuscript is well-structured, certain areas need refinement, including clarity in methodology, statistical analysis, and language consistency. I have provided detailed feedback and suggestions to enhance the rigour and readability of your work. I commend your efforts and look forward to your revised submission.

**General Comments**

  The manuscript presents an important study on the effectiveness of a mobile health program aimed at improving the physical and psychological capabilities of HIV voluntary counselling and testing (VCT) among the deaf community. While the topic is highly relevant, the manuscript requires several refinements to enhance clarity, methodology, statistical rigour, and language coherence. The following detailed comments guide areas that need improvement and offer examples of how revisions can be implemented.

**Title and Abstract:**

  The title of the manuscript should be refined to be more precise and informative. For instance, instead of *'The study protocol for a quasi-experimental study on the effectiveness of a mobile health program in enhancing the physical and psychological capabilities of HIV voluntary counseling and testing among the deaf community,'* a more concise version could be *'A Quasi-Experimental Study Protocol on the Impact of a Mobile Health Program for HIV Counseling and Testing in the Deaf Community.'* This revision ensures clarity and a direct reflection of the study’s scope.

 The abstract lacks specificity in the methodology section. Instead of stating, *'Quasi-experimental non-randomized controlled trial with single blinded participants,'* it would be clearer to write, *'This study employs a quasi-experimental design with a non-randomized controlled trial, involving single-blinded participants and a parallel group assignment.'* This revision provides more structure and readability.

**Introduction:**

 The introduction provides a strong background on the health challenges faced by the deaf community. However, it does not explicitly state the significance of the study. It would be beneficial to clearly articulate the research gap by including a statement like: *'Despite the increasing use of mobile health interventions in marginalized communities, there is limited evidence on their effectiveness in improving HIV-related health outcomes among deaf individuals. This study aims to bridge this gap by assessing a mobile health educational program specifically designed for this population.'* This addition will strengthen the rationale for the study.

 Additionally, some references cited in the introduction are outdated. Replacing them with more recent studies on digital health interventions will enhance the credibility of the literature review.

**Methods:**

  The study design should be described with greater detail. Instead of simply stating, *'Quasi-experimental non-randomized controlled trial with single blinded participants,'* a more comprehensive description could be: *'This quasi-experimental study follows a non-randomized controlled trial design, in which participants are assigned to either the intervention group, receiving the mobile health KaPi program, or the control group, receiving standard educational materials in the form of e-books. Participants will be blinded to the intervention allocation to minimize bias.'*

 The sample size calculation lacks clarity and should be expanded. The authors mention the use of G *Power software, but they do not provide the effect size used in the calculation. A more detailed explanation could be: 'Using GPower 3.1, the sample size was determined based on an expected medium effect size of 0.30, a power of 0.95, and an alpha level of 0.05. These parameters resulted in a required sample size of 80 participants, with 40 in each group.'*

 The intervention details are somewhat vague. Instead of stating, *'The KaPi mobile health program consists of 11 sessions,'* it would be more informative to describe: *'The KaPi mobile health program consists of 11 structured sessions, each lasting approximately 12 minutes. These sessions cover topics such as HIV prevention strategies, coping mechanisms for psychological distress, and self-efficacy improvement, all delivered through Indonesian sign language videos within the app.'* Providing this level of detail will help readers understand the intervention’s structure and expected impact.

**Ethical Considerations:**

  While ethical approval is mentioned, the manuscript should elaborate on the informed consent process. Instead of stating, *'Participants provided consent,'* it would be clearer to describe: *'All participants will be provided with a detailed study information sheet in Indonesian sign language, and informed consent will be obtained through video-recorded agreements to ensure accessibility for deaf individuals.'* This revision ensures that the ethical considerations specific to the deaf community are well addressed.

**Results Section (Planned Analysis):**

  The statistical analysis section should provide more depth on how changes over time will be measured. Instead of simply stating, *'The data will be analyzed using generalized estimation equations (GEE) with a confidence interval of 95%,'* a more precise description would be: *'The primary outcomes will be analyzed using generalized estimation equations (GEE) to account for within-group correlations over time. The model will include an interaction term between time and intervention to assess changes in psychological distress, HIV prevention practices, and self-efficacy levels. Adjusted odds ratios with 95% confidence intervals will be reported.'* This level of detail ensures clarity in the planned statistical approach.

**Discussion (Expected Implications):**

 The discussion should emphasize the study’s contributions to HIV prevention strategies for the deaf community. Instead of a general statement like, *'This study will help improve health outcomes among the deaf population,'* a more detailed perspective could be: *'By demonstrating the effectiveness of a culturally tailored mobile health intervention, this study has the potential to inform future digital health policies aimed at increasing HIV awareness and mental health support among deaf individuals.'*

 Potential limitations should also be discussed more explicitly. Instead of stating, *'This study may face some limitations,'* the authors could write, *'One limitation of this study is the reliance on self-reported measures, which may introduce reporting bias. Future studies should consider incorporating objective measures, such as biomarker data, to validate self-reported behavioral changes.'* This approach strengthens the critical evaluation of the study design.

**Language and Formatting:**

 The manuscript contains numerous grammatical errors and unclear sentences, which affect readability. For example, the sentence *'Deaf person is risk population for health'* should be revised to *'Deaf individuals are considered a high-risk population for health disparities.'* Careful proofreading and language editing will significantly improve the overall clarity and professionalism of the manuscript.

 Additionally, consistency in terminology is essential. Terms like *'deaf persons'* and *'hearing loss individuals'* should be standardized. Using *'deaf individuals'* throughout the text would improve coherence.

**Conclusion:**

 The conclusion should emphasize the expected contributions of the study rather than presenting definitive results. Instead of *'The intervention group is expected to show significant improvements in psychological distress and HIV prevention,'* a more appropriate statement would be: *'This study aims to provide evidence on the feasibility and effectiveness of a mobile health intervention tailored for deaf individuals, potentially informing future public health interventions and digital health strategies.'*

**Final Recommendation**

 The manuscript presents a valuable study but requires significant revisions for clarity, methodological rigor, and readability. Addressing the points outlined above will enhance the quality and impact of the research, ensuring that it effectively communicates its contributions to the field.

Is the study design appropriate for the research question?

Yes

Is the rationale for, and objectives of, the study clearly described?

Partly

Are sufficient details of the methods provided to allow replication by others?

Yes

Are the datasets clearly presented in a useable and accessible format?

Not applicable

Reviewer Expertise:

Public health, disease modelling, maternal and child health, HIV, Malaria, Neglected tropical diseases, Machine learning, communicable diseases, Global Health, mental health

I confirm that I have read this submission and believe that I have an appropriate level of expertise to confirm that it is of an acceptable scientific standard, however I have significant reservations, as outlined above.

# Response

**Published:** 2025-03-20

**Authors:** Yafi Sabila Rosyad

**Affiliations:** Nursing, Lincoln University College, Petaling Jaya, Selangor, Malaysia

1 I have revised the title of the article according to the reviewer's suggestion, which was originally titled The study protocol for a quasi-experimental study on the effectiveness of a mobile health program in enhancing the physical and psychological capabilities of HIV voluntary counseling and testing among the deaf community to A Quasi-Experimental Study Protocol on the Impact of a Mobile Health Program for HIV Counseling and Testing in the Deaf Community.

 2 abstract has little revision in methodology 

 3 statement at introduce "Despite the increasing use of mobile health interventions in marginalized communities, there is limited evidence on their effectiveness in improving HIV-related health outcomes among deaf individuals. This study aims to bridge this gap by assessing a mobile health educational program specifically designed for this population. By focusing on this underserved population, the research seeks to contribute valuable insights into the psychological distress, HIV prevention practice, and coping self-efficacy of deaf individuals, ultimately fostering a more inclusive approach to health care.

 4 correction Grammarly.
